# Latent variable mixture models to test for differential item functioning: a population-based analysis

**DOI:** 10.1186/s12955-017-0674-0

**Published:** 2017-05-15

**Authors:** Xiuyun Wu, Richard Sawatzky, Wilma Hopman, Nancy Mayo, Tolulope T. Sajobi, Juxin Liu, Jerilynn Prior, Alexandra Papaioannou, Robert G. Josse, Tanveer Towheed, K. Shawn Davison, Lisa M. Lix

**Affiliations:** 1 School of Public Health, University of Alberta, Edmonton, AB Canada; 2 School of Public Health and Health Management, Weifang Medical University, Weifang, Shandong Province China; 3 School of Nursing, Trinity Western University & Centre for Health Evaluation and Outcomes Sciences, Providence Health Care, Langley, BC Canada; 4 Department of Public Health Sciences, Queen’s University, Kingston, ON Canada; 5 McGill University Health Centre, Montréal, QC Canada; 6 Department of Community Health Sciences & O’Brien Institute for Public Health, University of Calgary, Calgary, AB Canada; 7 Department of Mathematics and Statistics, University of Saskatchewan, Saskatoon, SK Canada; 8 Division of Endocrinology, Department of Medicine, University of British Columbia, Vancouver, BC Canada; 9 Department of Medicine, McMaster University, Hamilton, ON Canada; 10 Department of Medicine, Faculty of Medicine, University of Toronto, Toronto, ON Canada; 11 Department of Medicine, Queen’s University, Kingston, ON Canada; 12 Faculty of Graduate Studies, University of Victoria, Victoria, BC Canada; 13 Department of Community Health Sciences, University of Manitoba, S113-750 Bannatyne Ave, Winnipeg, MB R3E 0W3 Canada

**Keywords:** Latent class analysis, Item response theory, Mental health, Patient-reported outcome measures, Physical functioning, Population health

## Abstract

**Background:**

Comparisons of population health status using self-report measures such as the SF-36 rest on the assumption that the measured items have a common interpretation across sub-groups. However, self-report measures may be sensitive to differential item functioning (DIF), which occurs when sub-groups with the same underlying health status have a different probability of item response. This study tested for DIF on the SF-36 physical functioning (PF) and mental health (MH) sub-scales in population-based data using latent variable mixture models (LVMMs).

**Methods:**

Data were from the Canadian Multicentre Osteoporosis Study (CaMos), a prospective national cohort study. LVMMs were applied to the ten PF and five MH SF-36 items. A standard two-parameter graded response model with one latent class was compared to multi-class LVMMs. Multivariable logistic regression models with pseudo-class random draws characterized the latent classes on demographic and health variables.

**Results:**

The CaMos cohort consisted of 9423 respondents. A three-class LVMM fit the PF sub-scale, with class proportions of 0.59, 0.24, and 0.17. For the MH sub-scale, a two-class model fit the data, with class proportions of 0.69 and 0.31. For PF items, the probabilities of reporting greater limitations were consistently higher in classes 2 and 3 than class 1. For MH items, respondents in class 2 reported more health problems than in class 1. Differences in item thresholds and factor loadings between one-class and multi-class models were observed for both sub-scales. Demographic and health variables were associated with class membership.

**Conclusions:**

This study revealed DIF in population-based SF-36 data; the results suggest that PF and MH sub-scale scores may not be comparable across sub-groups defined by demographic and health status variables, although effects were frequently small to moderate in size. Evaluation of DIF should be a routine step when analysing population-based self-report data to ensure valid comparisons amongst sub-groups.

## Background

Self-report health status measures, such as the SF-36, are frequently used to compare the health and well-being of different populations and to establish population norms for comparative investigations [1]. To make accurate comparisons, the scores on these patient-reported outcome measures (PROMs) must be valid and reliable. While many PROMS have been subjected to recommended psychometric evaluations of their construct validity, test-retest reliability, and interpretability [2–4], they are less frequently evaluated for differential item functioning (DIF), a form of measurement non-equivalence. Specifically, measurement non-equivalence (or non-invariance) exists when a PROM does not measure the same construct (e.g., quality of life) across different population sub-groups [5, 6]. DIF arises when individuals with the same underlying level of health respond differently to the items that comprise a health measure; these differences are often associated with respondent characteristics such as sex and age [7]. If there is no evidence of DIF, individuals with the same characteristics are expected to produce a similar pattern of responses on the PROM items. However, when there is evidence of DIF, differences on the PROM items may be an artifact of the measurement process.

Measurement non-equivalence and DIF have been examined for the SF-36 and SF-12 in population-based data [8–12]. DIF has been detected in both instruments [8, 10–12].

Item response theory (IRT) models, which were originally developed for application to achievement tests in the field of education and psychology, are commonly used to test for DIF [13] amongst manifest (i.e., observed) groups, such as males and females. However, comparisons of item responses for manifest groups using conventional IRT models may lack sensitivity to detect the true source(s) of DIF [14–17]. Specifically, DIF may be associated with unobserved (i.e., latent) characteristics, as well as observed characteristics. Alternative analytic techniques to conventional IRT models are needed to disentangle the effects of latent and observed characteristics [14–17].

Latent variable mixture models (LVMMs) have recently been proposed to detect DIF-related bias in self-report measures [10, 14–21]. LVMMs combine latent class techniques with IRT; these models do not assume that manifest variables fully account for differential patterns of item functioning [15, 16]. A LVMM for DIF seeks to identify heterogeneous latent groups (i.e., latent classes) in a population with distinct patterns of item responses. The LVMM assumes that the IRT measurement model holds within each latent class, but allows for different sets of item parameters among the latent classes. LVMMs are well suited to the analysis of population-based data, which are likely to exhibit substantial heterogeneity in patterns of item responses.These models are valuable because they can be used to generate hypotheses about population characteristics that may be associated with DIF [10].

The purpose of this study was to test for DIF on self-report measures of physical and mental health in a diverse population-based cohort using LVMMs. Specifically, we focus on the SF-36 physical functioning (PF) and mental health (MH) sub-scales. These sub-scales were selected for investigation because previous studies have demonstrated their sensitivity to DIF using manifest variable models [8, 9, 11, 22, 23]. An investigation of whether LVMMs will detect DIF in these two SF-36 sub-scales can help to elucidate the causes of DIF and facilitate the interpretation of DIF and its impact on the validity of the SF-36.

## Methods

### Data source

Study data were from the Canadian Multicentre Osteoporosis Study (CaMos), a population-based prospective cohort study that was initiated to provide unbiased national estimates of the prevalence and incidence of osteoporosis [24]. Individuals were recruited without regard for disease status but had to live within a 50-km radius of one of the nine study sites, which were primarily in urban centres. Baseline data, which were the focus of this analysis, were collected in 1996 to 1997. Details of the data collection methods and sample characteristics have been published elsewhere [24]. We included respondents 25 years of age and older.

### Measures

The SF-36 version 1 was used in the CaMos; it encompasses eight sub-scales: PF, MH, role physical, bodily pain, general health, vitality, social functioning, and role emotional. The PF sub-scale is comprised of 10 items with three response options: *limited a lot*, *limited a little*, and *not limited at all*. The MH sub-scale is comprised of five items evaluated on a six-point scale with endpoints of *all of the time* and *none of the time* [1, 25].

The study cohort was described on the demographic variables of sex, age, and education level, and on the health status variables of self-reported general health status, measured height and weight from which body mass index (BMI) was computed, and health utilities. The latter were measured via the Health Utilities Index Mark 3 (HUI3), a preference-based measure that captures functional performance in vision, hearing, speech, ambulation, dexterity, emotion, cognition, and pain [26]. HUI3 summary scores were estimated using a multi-attribute utility function [27]; a score of zero corresponds to death and 1.00 corresponds to a complete absence of impairment. Negative scores are possible and correspond to a severely impaired health state worse than death.

### Statistical Analyses

Descriptive statistics were used to characterize the study sample on the PF and MH item responses, demographic, and health status measures**.** Item responses with positively worded formats (i.e., MH3 and MH5) were reverse coded prior to analysis.

Analyses were conducted separately for the PF and MH sub-scale items. Unidimensionality, the assumption that all items measure a single construct, was investigated by a descriptive analysis of the standardized residuals of item responses for the one-class model [28]. A *χ* ^2^ statistic was used to test for differences between predicted and observed responses. We also examined unidimensionality using exploratory factor analysis with oblique rotation applied to the polychoric correlations for the items [29]. The root mean square error of approximation (RMSEA) and comparative fit index (CFI) were used to assess model fit. A RMSEA value ≤0.10 [30], and a CFI value >0.90 [31] indicate acceptable model fit.

Local independence of the items is satisfied if the residuals of items are not correlated, conditional on the common latent factor (i.e., P[R_1_, R_2_ | θ] = P[R_1_| θ] P[R_2_| θ] where θ represents underlying health status and R_1_ and R_2_ are residuals for scale items 1 and 2. In other words, the joint probability of correct responses for an item pair will be a product of the probabilities of correct responses to the two items, conditional on the latent factor. We evaluated local independence by comparing the predicted and observed proportions of responses for each pair of items in a sub-scale in all the models using a χ^2^ statistic [32].

The LVMM combines a latent class model with an IRT two-parameter graded response model (GRM) [33]. This GRM is specified as:

1 $$ {P}_{i j\left({X}_i\ge j|\theta, {a}_i\right)}=\frac{ \exp\ \left[{\alpha}_{i\ }\left(\theta -{\beta}_{i j}\right)\right]}{1+ \exp\ \left[{\alpha}_{i\ }\left(\theta -{\beta}_{i j}\right)\right]}, $$

where *P*_*ij*_ is the cumulative probability that a person receives a score on the *j*th or higher category (*j* = 1, 2,…, *J*) for item *i* (*i* = 1, …, *I*). In Eq. 1, *θ* represents underlying health status, *β*_*ij*_ is the item response parameter (also referred to as the difficulty parameter) for category *j* or above relative to lower categories and *α*_*i*_ is the item discrimination parameter (i.e., slope) for item *i* indicating the relationship between the item and the latent construct [33].

We adopted Muthén’s LVMM framework [34], which estimates factor loadings (*λ*) and item thresholds (*τ*); these can be converted to item discrimination and difficulty parameters [35]. The item factor loadings and thresholds in the LVMM are allowed to vary across two or more latent classes (see Figure in Appendix).

The conditional cumulative probability of the item response within the *m*th latent class is estimated by.

2 $$ {P}_{i jm\left({X}_i\ge j|\theta, C= m\right)}=\frac{ \exp \left({\lambda}_{i m\ }\theta -{\tau}_{i jm}\right)}{1+ \exp \left({\lambda}_{i m\ }\theta -{\tau}_{i jm}\right)\ } $$

In Eq. 2, *C* denotes the latent class variable (*m =* 1*,…, M*), *λ*_*im*_ is the estimated factor loading for item *i* within class *m*, and *τ*_*ijm*_ is the estimated threshold for response categories at or above category *j* for item *i* within class *m*. The cumulative probability of an item response for category *j* and higher for a respondent is estimated by:

3 $$ {P}_{i j}={\sum}_{m=1}^M\left({\pi}_m\times {P}_{i j m}\left({X}_i\ge j|\theta \right)\right), $$

where *π*_*m*_ is the posterior probability for a respondent belonging to the *m*th latent class [35]. The posterior probability was estimated using Bayes’ theorem [36]. Respondents were assigned to the latent class with the largest posterior probability among the classes.

To examine DIF and its impact on the underlying latent health variable (i.e., PF or MH), we adopted a four-step procedure [10]: (a) fit the standard IRT two-parameter GRM (i.e., one-class model), and multiple-class LVMMs to identify the best-fit model, (b) test for differences in the model parameters across the latent classes, (c) characterize potential sources of DIF by comparing within-class item response percentages and test associations of class membership with the covariates, and (d) examine the impact of DIF by comparing predicted factor scores between the one-class GRM and the multiple-class LVMM.

A one-class model was compared to two-, three-, and four-class models to determine the optimal number of latent classes. The statistics used to select the best-fit model included the Bayesian Information Criterion (BIC) [37, 38], Vuong-Lo-Mendell-Rubin likelihood ratio test (VLMR) [39] and bootstrap likelihood ratio test (BLRT) [37]. A good-fitting multi-class model with estimated class-specific item parameters provides evidence of latent DIF [20]. Quality of class assignment was evaluated with the entropy measure, which ranges between 0 and 1; larger values indicate a higher proportion of correct classifications [40]. The maximum likelihood method with robust standard errors was used to estimate the model parameters [29]. In order to identify the model, the latent factor mean was set to zero and the variance was set to one across classes [34], which is in accordance with conventional IRT parameterization for DIF [34, 41, 42]. The model allowed the item parameters to vary across latent classes.

The differences in threshold parameters amongst the latent classes were tested for each item using a likelihood ratio (LR) test [37, 43]. Specifically, a model with free threshold parameters for an item was compared with a model in which the threshold parameters were constrained to be equal across classes for an item. A statistically significant difference between the two models indicates uniform DIF for the item [7].

Multinomial logistic regression with pseudo-class random draws was used to characterize the latent classes on selected demographic and health status variables. Age was categorized as younger (25–64 years) and older (65+ years). BMI was categorized as overweight and obese (BMI ≥ 25.0) versus underweight and normal weight (BMI < 25.0) [44]. Based on the distribution of the HUI3, scores were categorized as low (<0.8) and high (≥0.8). Odds ratios (ORs) and 95% confidence intervals (95% CIs) were reported.

Predicted factor scores and the most likely class membership for each respondent were obtained from the best-fitting LVMM. The reliability of the predicted factor scores was evaluated by the conditional standard error of measurement (CSEM), which was calculated as the inverse of the square root of the information function [45]. Skewness was examined for the distribution of the predicted factor scores [46].

SAS 9.3 was used to prepare the data and conduct the descriptive analyses [47]. The LVMM analysis was conducted with Mplus version 7.11 [29].

## Results

### Description of cohort

The CaMos cohort was comprised of 9423 respondents. In total, 9337 (99.1%), and 9395 respondents (99.7%) had complete data on the 10 PF items and five MH items, respectively.

For the PF items, close to half (43.9%) of respondents reported being limited a lot in vigorous activities, while less than 6.0% of respondents reported being limited a lot when climbing one flight of stairs, walking one block, or bathing/dressing (Table 1). For the MH items, less than 2.0% of respondents reported that they were nervous, so down in the dumps that nothing could cheer them up, or feeling downhearted and blue all of the time.

**Table 1:** Distribution of item responses (%) for the SF-36 sub-scale items

Item	Response option					
Physical Functioning (*N* = 9337)	Limited a lot	Limited a little		Not limited at all		
PF1: Vigorous activities	43.9	34.2		21.9		
PF2: Moderate activities	12.7	24.4		62.9		
PF3: Lifting or carrying groceries	9.1	22.2		68.8		
PF4: Climbing several flights of stairs	15.2	29.6		55.1		
PF5: Climbing one flight of stairs	5.6	15.8		78.6		
PF6: Bending, kneeling or stooping	13.0	33.0		54.0		
PF7: Walking more than a mile	16.5	20.6		62.9		
PF8: Walking several blocks	10.8	15.0		74.2		
PF9: Walking one block	4.0	9.9		86.0		
PF10: Bathing or dressing self	2.0	6.5		91.5		
Mental Health (*N* = 9395)	All of the time	Most of the time	Good bit of the time	Some of the time	Little of the time	None of the time
*Have you…*						
MH1: Been a very nervous person?	1.3	3.1	5.4	17.2	29.7	43.3
MH2: Felt so down in the dumps that nothing could cheer you up?	0.3	1.1	1.8	7.4	17.0	72.4
MH3: Felt calm and peaceful?	10.6	46.3	18.2	15.7	5.9	3.2
MH4: Felt downhearted and blue?	0.4	1.6	3.4	17.8	34.8	42.1
MH5: Been a happy person?	18.5	54.8	12.9	8.7	2.9	2.3

### Examination of unidimensionality and local independence

For the PF sub-scale items, the IRT two-parameter GRM fit the data well as evidenced by absolute values of standardized residuals less than 1.96 for all items. The difference between observed and predicted proportions of item responses was not statistically significant (χ^2^ = 9.01, df = 27, *p* = 0.999), suggesting that the 10 items represented a single construct.

Confirmatory factor analysis for a single latent construct resulted in RMSEA = 0.11 and CFI = 0.98, with the former indicating poor model fit. A confirmatory factor analysis model with error covariances between five pairs of items (PF1 and PF2, PF2 and PF3, PF4 and PF5, PF7 and PF8, PF8 and PF9) resulted in a good fit (RMSEA = 0.057, CFI = 0.99). Thus, the factor structure suggested a single latent factor solution for the PF scale items. Pairwise comparisons of predicted and observed proportions of item responses for all items revealed a poor fit to the data (χ^2^ = 3020.96, df = 351, *p* < 0.001), suggesting that the assumption of local independence might not be tenable and heterogeneous subgroups might exist in the sample [16, 48].

For the MH sub-scale, the IRT two-parameter GRM fit the data well with respect to a unidimensional model. Absolute values of standardized residuals were less than 1.96 for all sub-scale items. Univariate model fit statistics indicated a non-significant difference between observed and predicted item responses (χ2 = 6.61, df = 29, *p* = 0.999). Confirmatory factor analysis revealed that one-factor model was not a good fit as judged by the RMSEA = 0.15, although the CFI (0.97) indicated good fit. These results support a single dominant latent factor for both the PF and MH sub-scale items. However, a statistically significant difference in the joint distributions of item pairs (χ2 = 1952.12, df = 159, *p* < 0.001) for MH sub-scale was also observed, suggesting that local dependence might exist, and thus a mixture IRT model with more than one class might be a better choice to account for local dependence.

### Fitting models with latent classes

For the PF sub-scale, the three-class LVMM had a better fit than the one- and two-class models; the BIC, VLMR and BLRT all favored the three-class model (Table 2). For the MH sub-scale, the two-class model yielded better fit indices than the one-class GRM. Therefore, three- and two-class models were chosen as the best-fitting models for the PF and MH sub-scales, respectively (Table 2).

**Table 2:** Model fit statistics and class proportions for latent variable mixture models on the SF-36 physical functioning and mental health sub-scale items

# Classes	# Par	BIC	VLMR *p*-value	BLRT *p*-value	Entropy	Class proportions^a^			
						Class 1	Class 2	Class 3	Class 4
Physical Functioning									
1	28	102,298.56				1.00			
2	57	100,569.46	<0.0001	<0.0001	0.55	0.61 (0.68)	0.39 (0.32)		
**3**	86	**100,083.64**	**0.0007**	**<0.0001**	**0.64**	**0.59 (0.64)**	**0.24 (0.22)**	**0.17 (0.14)**	
4	115	99,806.68	NA	NA	0.60	0.51 (0.55)	0.22 (0.19)	0.17 (0.16)	0.10 (0.10)
Mental Health									
1	20	95,536.81				1.00			
**2**	41	**94,631.65**	**<0.0001**	**<0.0001**	**0.40**	**0.69 (0.78)**	**0.31 (0.22)**		
3	62	94,363.51	NA	NA	0.44	0.25 (0.20)	0.37 (0.40)	0.38 (0.40)	

Bold values indicate the model that was selected for further analysis. # Par = number of parameters. *BIC* Bayesian information criterion. *VLMR* Vuong-Lo-Mendell-Rubin likelihood ratio test. *BLRT* Bootstrap likelihood ratio test. *NA* Not Applicable

^a^Classification of individuals based on their most likely latent class is shown in parentheses

For the PF sub-scale items, threshold values in class 2 were uniformly larger than in class 1. As well, there were larger threshold values amongst respondents in class 3 than in class 2. This indicates that respondents with the same level of latent ability had higher probabilities of endorsing lower categories for items representing limited PF in class 2 and class 3 relative to class 1. Differences in factor loadings were found for the items across the classes, suggesting that items varied in their ability to differentiate amongst respondents.

For the MH sub-scale, the absolute value of differences in item thresholds between the two classes ranged from 0.38 for MH1 to 3.56 for MH5. Factor loadings for class 2 were smaller than for class 1.

Table 3 shows the LR statistics to test for DIF on PF and MH sub-scale items. Overall, nine of the 10 items for the PF sub-scale and all the five items in the MH sub-scale showed uniform DIF.

**Table 3:** Likelihood ratio (LR) test statistics for differential item functioning on the SF-36 physical functioning and mental health sub-scale items

Item	LR Statistic	Δdf^*^	*p*-value
Physical Functioning			
PF1: Vigorous activities	99.63	4	<0.0001
PF2: Moderate activities	127.49	4	<0.0001
PF3: Lifting or carrying groceries	151.04	4	<0.0001
PF4: Climbing several flights of stairs	37.69	4	<0.0001
PF5: Climbing one flight of stairs	60.95	4	<0.0001
PF6: Bending, kneeling or stooping	141.85	4	<0.0001
PF7: Walking more than a mile	87.79	4	<0.0001
PF8: Walking several blocks	34.51	4	<0.0001
PF9: Walking one block	3.26	2	0.196
PF10: Bathing or dressing self	64.76	2	<0.0001
Mental Health			
MH1: Been a very nervous person?	71.79	3	<0.0001
MH2: Felt so down in the dumps that nothing could cheer you up?	21.03	3	0.0001
MH3: Felt calm and peaceful?	234.43	3	<0.0001
MH4: Felt downhearted and blue?	52.27	3	<0.0001
MH5: Been a happy person?	198.28	3	<0.0001

LR statistics are for the comparison of nested models: (a) mixture IRT model with free thresholds across classes for each item, and (b) IRT model with constrained thresholds across classes for each item. The mixture IRT model accounted for local dependence. Note that the latent variable mixture model for the physical functioning sub-scale had 3 classes and the model for the mental health sub-scale had 2 classes. Δdf is the difference in degrees of freedom for models defined in (a) and (b)

### Sources of DIF

**Fig. 1:**
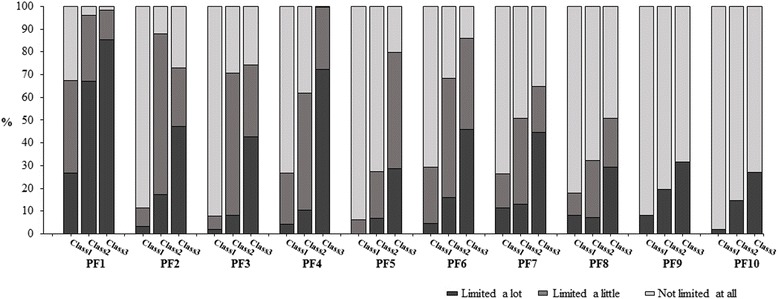
Within-class item response percentages for the SF-36 physical functioning (PF) sub-scale items. Legend: The selected best-fit latent variable mixture model had three classes. For items PF9 and PF10, response categories of “limited a lot” and “limited a little” were combined due to low frequencies

Figure 1 compares the response percentages for each of the three latent classes on the PF sub-scale items. The prevalence of PF limitations was significantly higher in classes 2 and 3 compared to class 1. Overall, 98.2% of respondents reported having limitations in class 3 on “vigorous activities”compared to only 67.4% for class 1. As well, 99.8% of respondents had limitations on “climbing several flights of stairs” in class 3 compared to only 26.7% in class 1.

**Fig. 2:**
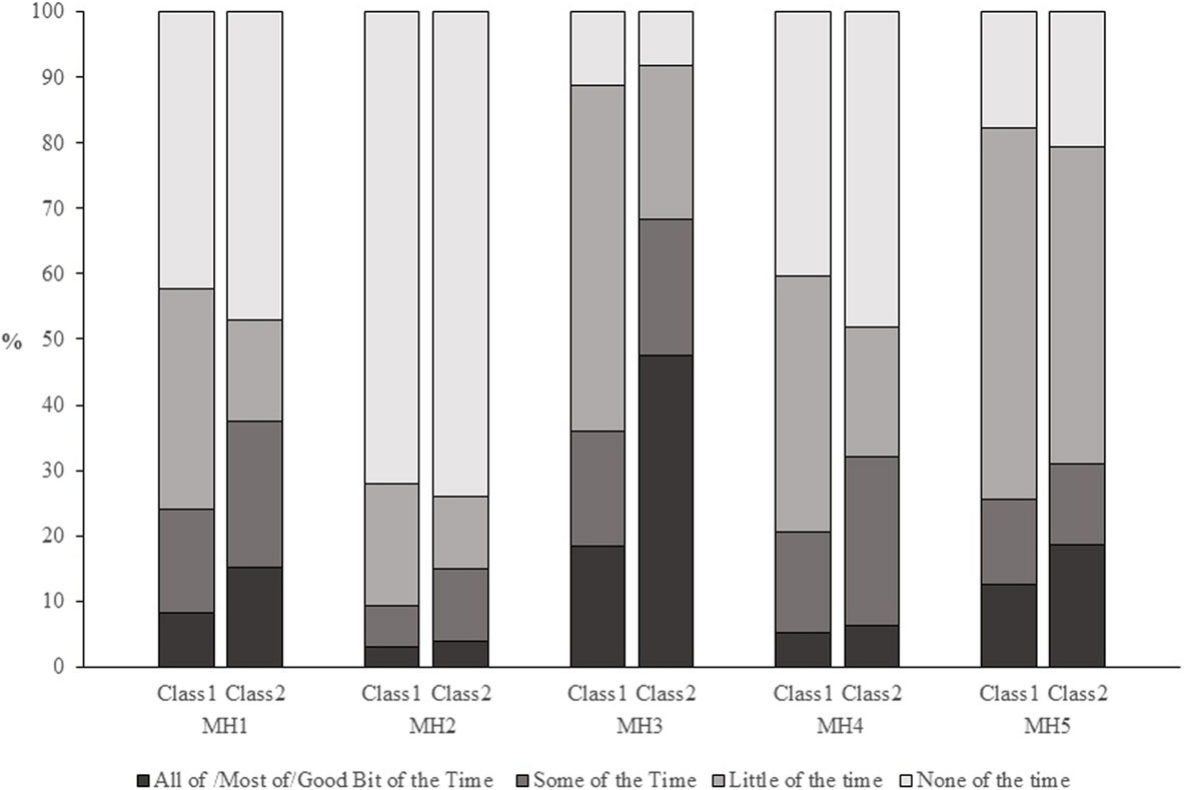
Within-class item response percentages for the SF-36 mental health (MH) sub-scale items. Legend: The selected best-fit latent variable mixture model had two classes. Response categories of “all of the time”, “most of the time” and “good bit of the time” were combined due to low frequencies. MH3 and MH5 were reverse coded in the analysis

Similarly, respondents reported more MH problems in class 2 than class 1 (Fig. 2). For example, 15.2% of respondents in class 2 reported having been a very nervous person (MH1) all of the time/most of the time/a good bit of the time, compared to just 8.3% of respondents in class 1. Close to half (47.4%) of respondents in class 2 reported feeling calm and peaceful none of the time/little of the time/some of the time relative to only 18.5% of respondents in class 1.

Table 4 contains the results for the logistic regression analysis of the associations between latent class membership and the demographic and health status variables. For the three classes for the PF sub-scale items, the odds of class membership were higher for females, older respondents, those with a lower level of general health status, and lower HUI3 index scores in both of classes 2 and 3, relative to class 1. Respondents with lower educational attainment (i.e., less than high school), and who were overweight/obese had a greater odds of being in class 3 than in class 1. For the MH sub-scale, the logistic model revealed that respondents of older age, with less than high school education, and good health had a greater odds of being in class 2 than class 1.

**Table 4:** Distribution (%) and odds ratios (ORs) for demographic and health status characteristics by latent class

	Distribution (%)			OR (95% CI)	
	Class 1	Class 2	Class 3	Class 2	Class 3
Physical Functioning					
Class Frequencies	5933	2081	1323		
Sex					
Men	35.5	23.4	20.6	Ref	Ref
Women	64.5	76.6	79.4	**1.66** (1.44, 1.91)	**1.76** (1.48, 2.10)
Age					
25–64 years	60.5	40.7	31.0	Ref	Ref
≥ 65 years	39.5	59.3	69.0	**1.69** (1.48, 1.92)	**1.81** (1.55, 2.12)
Education					
Postsecondary	53.7	43.7	33.0	Ref	Ref
High school graduate	14.9	14.0	13.8	1.01 (0.84, 1.22)	1.11 (0.88, 1.38)
Less than high school	31.4	42.4	53.2	1.13 (0.98, 1.30)	**1.32** (1.12, 1.56)
General Health					
Excellent/Very good	68.1	42.0	30.0	Ref	Ref
Good	26.3	43.2	40.7	**1.99** (1.73, 2.29)	**1.86** (1.58, 2.18)
Fair/Poor	5.6	14.8	29.3	**2.42** (1.94, 3.01)	**3.53** (2.78, 4.47)
Weight Status					
Underweight/Normal	37.1	39.3	31.9	Ref	Ref
Overweight/Obese	62.9	60.7	68.1	0.99 (0.87, 1.12)	**1.21** (1.04, 1.41)
HUI3					
High (≥0.8)	81.1	58.9	38.3	Ref	Ref
Low (<0.8)	18.9	41.1	61.8	**1.92** (1.67, 2.22)	**2.62** (2.22, 3.09)
Mental Health					
Class Frequencies	7337	2058			
Sex					
Men	30.9	29.7		Ref	
Women	69.1	70.3		1.03 (0.90, 1.17)	
Age					
25–64 years	53.8	44.8		Ref	
≥ 65 years	46.3	55.2		**1.18** (1.05, 1.33)	
Education					
Postsecondary	50.2	42.4		Ref	
High school graduate	14.6	14.1		1.09 (0.91, 1.30)	
Less than high school	35.2	43.4		**1.21** (1.06, 1.38)	
General Health					
Excellent/Very good	58.7	49.8		Ref	
Good	31.1	36.5		**1.13** (1.00, 1.28)	
Fair/Poor	10.3	13.8		1.17 (0.95, 1.43)	
Weight Status					
Underweight/Normal	37.4	34.8		Ref	
Overweight/Obese	62.6	65.2		1.07 (0.96, 1.21)	
HUI3					
High (≥0.8)	71.5	65.0		Ref	
Low (<0.8)	28.5	35.0		1.01 (0.89, 1.16)	

95% CI = 95% confidence interval; HUI3 = Health Utilities Index Mark 3; bold values indicate ORs that are statistically significant at α = .05. Class 1 is the reference group for both models. For the physical functioning sub-scale, the latent variable mixture model has three classes, while for the mental health sub-scale, the latent variable mixture model has two classes

### Impact of DIF on predicted factor scores

**Fig. 3:**
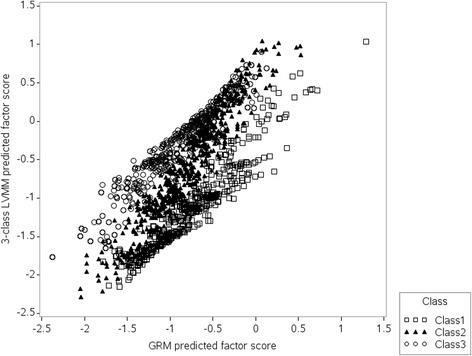
Predicted factor scores for the SF-36 physical functioning (PF) sub-scale. Legend: Predicted factor scores were for the one-class graded response model (GRM) and the three-class latent variable mixture model (LVMM)

For the PF sub-scale, factor scores for the standard one-class GRM and the three-class LVMM were strongly correlated (Pearson *r* = 0.89). Despite the high correlation, there were differences in the tails of the distributions. The standardized difference was ≤ −0.5 for 20.2% of the sample and ≥0.3 for 26.2% of the sample (Fig. 3).

**Fig. 4:**
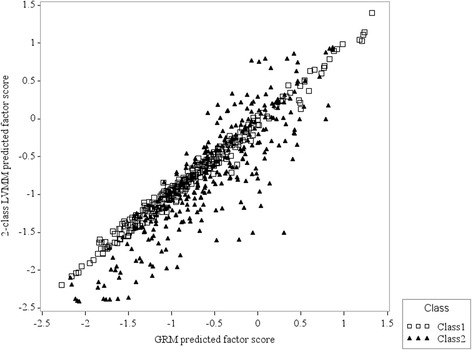
Predicted factor scores for the SF-36 mental health (MH) sub-scale. Legend: Predicted factor scores were for the one-class graded response model (GRM) and the two-class latent variable mixture model (LVMM)

For the MH sub-scale, differences in factor scores between the one-class and two-class models for the MH items were also observed. Specifically, 2.6% of respondents had a standardized difference ≥ 0.3 and 3.9% of respondents had a difference ≤ −0.3 (Fig. 4).

There were differences in the CSEMs between the one-class and multi-class models. For example, relative to the one-class model for the PF sub-scale items, in the three-class model there was greater measurement precision in the lower range of the theta distribution (i.e., theta <0) for class 1; greater measurement precision was observed in the upper range of the theta distribution (theta >0) for class 3.

## Discussion

Using a LVMM, this study identified three groups of individuals with different response patterns on the SF-36 PF sub-scale items and two groups of individuals with different response patterns on the MH sub-scale items in the population-based CaMos. For both sub-scale analyses, latent class 1 primarily contained respondents with fewer health limitations or problems. For the PF sub-scale, class 2 and 3 respondents were more likely to be female, older, and in poorer health than respondents in class 1. For the MH sub-scale, older respondents with lower education and good health were more likely to be in class 2. These findings indicate that DIF is present in unobserved groups, and the source of DIF can be at least partly explained by demographic and health status variables.

The results for the PF sub-scale are consistent with those from a previous study of the Canadian adult population [10], in which a three-class LVMM was found to fit the data better than one- or two-class models. We observed similar results in terms of item response patterns, effect of DIF on the latent factor scores, and the relationship between the latent class membership and selected socio-demographic and health variables. That previous study also found that the first latent class contained more people with no limitations in PF than the other two classes, and that sex, age and health status were associated with latent class membership. This provides support for our conclusion that DIF can affect responses to the SF-36 items and helps to validate the utility of the mixture model for exploring DIF.

For the MH sub-scale, we observed larger differences in both factor loadings and thresholds between the two latent classes for the following items: “Have you felt calm and peaceful?”, and “Have you been a happy person?”. The low values of the factor loadings for the two items in the second latent class suggests that the two items have low ability to discriminate between respondents having higher and lower levels of mental health. In other words, a unit change in the factor scores in one class is not associated with the same change in the factor scores in the other class [5]. Possible reasons may be the different wording format for these two items when compared to the other MH items. Previous studies have reported that combinations of negatively and positively worded questions may compromise internal consistency of measures [49, 50].

The LVMM offers a number of advantages for DIF detection relative to traditional IRT models that focus on DIF investigations for manifest variables. The standard IRT model assumes there is one homogeneous group (i.e. one latent class) of individuals within a population and DIF is examined for subgroups of the population based on manifest characteristics. However, true sources of DIF may not be limited to manifest groups (i.e., sex, age) conditional on their latent ability. The LVMM is optimal for DIF examination when the origin(s) of differential response patterns is not known a priori. Prior studies have revealed that the mixture IRT model is a good alternative for detecting DIF [15, 17].

In implementing the LVMM, a critical step is to identify the best-fitting model with an appropriate number of latent classes. A potential problem can arise when the model converges on a local solution rather than the expected global maximum likelihood solution, resulting in biased model parameters [51]. To test the stability of the parameter estimates for the multiple-class models, we performed analyses by fitting models with the same number of classes but with different numbers of random starts [29]. The parameter estimates from the models with different sets of random starts were very similar, suggesting a stable and robust result. Another critical step is to characterize the latent classes. To do this, we used a pseudo-class methodology [52, 53] that has been shown to produce unbiased parameter estimates [52, 54]. Inaccurate numbers of latent classes may result from misspecification of the model [55], or the distribution of the data [56]. However, simulation and empirical studies have demonstrated the sensitivity of the LVMM [14–16, 20, 21, 57–59] for DIF assesment. Use of LVMMs can aid researchers to identify and characterize DIF effects in self-reported health outcome measures.

In the context of testing for DIF using the LVMM, there has been discussion in the literature about the optimal approach to scale the latent variable(s) in order to enable comparisons of item parameters. One approach is to allow latent factor means and variances to vary in each class, with equality constraints for item thresholds for one or more class-invariant items (also called anchor items) [60]. For this method, anchor items must be identified. Another approach is to set the latent factor means to zero and variances to one across classes; then the item parameters (i.e., factor loadings or item thresholds) are freely estimated across classes. We adopted this latter approach (i.e., with unequal item thresholds across classes). We subsequently tested for DIF on each item by fitting a set models with constrained item thresholds between latent classes for each item one at a time. The method has been used in previous studies for DIF detection using LVMMs [20, 61].

The study limitations must also be acknowledged. We analysed only the SF-36 PF and MH sub-scale items. Other sub-scales may also exhibit DIF, but they contain small numbers of items and therefore are more difficult to analyze using LVMMs. The sample was comprised of a smaller proportion of men and more older adults than in the general Canadian population; the findings may therefore not be representative of younger people and men. Although we observed comparable findings regarding DIF effects for the PF sub-scale with previous research in Canada [9], further research is needed to determine whether our findings are applicable to populations from other countries. Strengths of the present study include the use of a large national population-based sample, and the ability to consider both demographic and health status variables to characterize the latent classes. Other health status variables, such as the presence of selected chronic conditions, might be considered, if supported by research.

## Conclusions

In conclusion, this study identified latent groups of respondents for whom the SF-36 PF and MH items function differently in a diverse national adult sample. The LVMM was a useful tool to define sub-groups of individuals with similar item response patterns and investigate characteristics potentially associated with group member. This analysis provides information that can be useful for generating hypotheses for future studies about DIF; for example, one might use these results to conduct DIF analyses for observed population sub-groups in independent samples. Testing for DIF should be a routine part of comparative analyses of population health status. The comparability of SF-36 sub-scale scores can be significantly compromised by heterogeneity in item responses, which can affect the interpretation of results from clinical and epidemiologic studies. Future study is needed to validate the present findings in other samples to inform generalizability of the results. Future research might also compare the sensitivity of the PF and MH sub-scales to detect DIF effects using both manifest variable and LVMM approaches.

## Figures and Tables

**Fig. 5:**
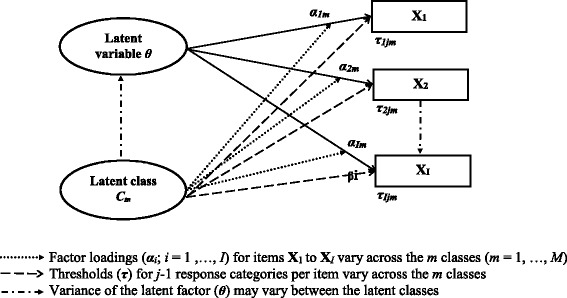
The latent variable mixture model (LVMM) with class-specific parameters
